# Multiple Brown Tumors in Primary Hyperparathyroidism

**DOI:** 10.1210/jcemcr/luae157

**Published:** 2024-08-27

**Authors:** Abrar Ali Chhachhar, Muhammad Qamar Masood

**Affiliations:** Section of Diabetes, Endocrinology, and Metabolism, Department of Medicine, Aga Khan University, Karachi 74800, Pakistan; Section of Diabetes, Endocrinology, and Metabolism, Department of Medicine, Aga Khan University, Karachi 74800, Pakistan

**Keywords:** brown tumors, hyperparathyroidism, parathyroidectomy, osteitis fibrosa cystica

## Image Legend

**Figure 1.**
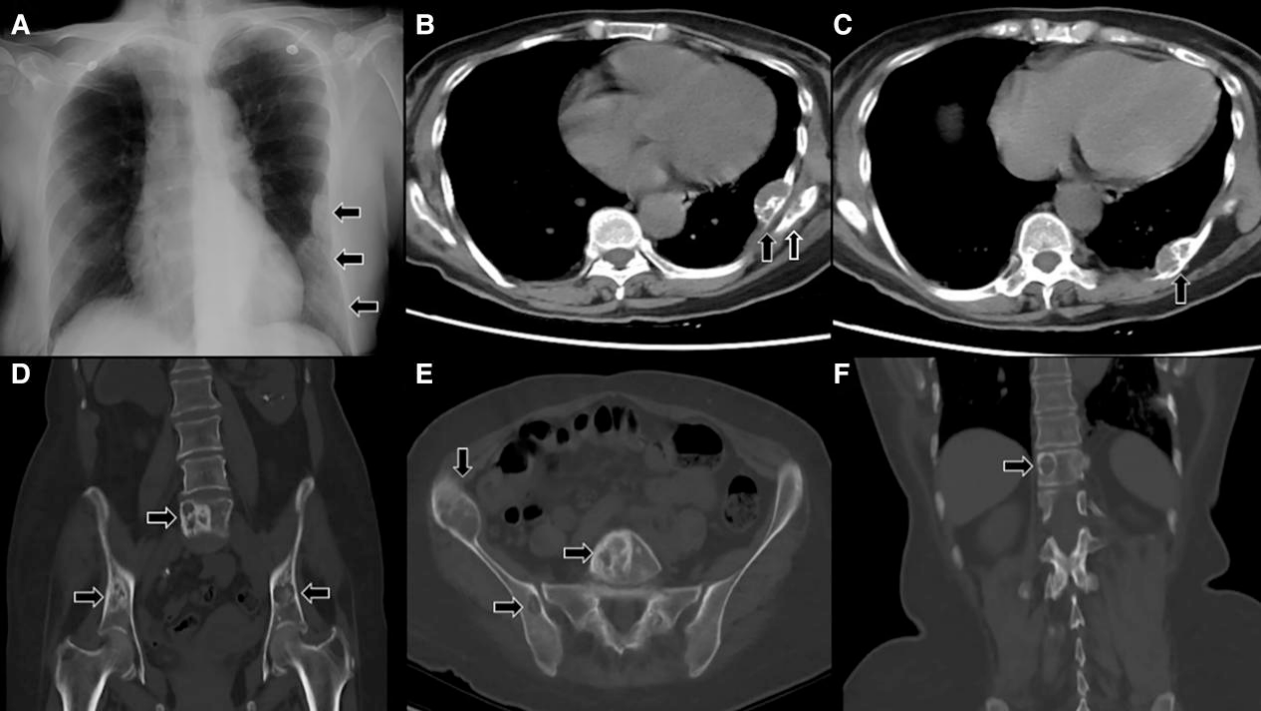
A, Pleural lesions involving eighth, ninth, and tenth ribs. B, Mixed sclerotic and lytic lesions with cortical expansion in the scapula and left ninth rib (39 × 15 mm) near angle of the rib. C, Mixed sclerotic and lytic lesion in left tenth rib (30 × 16.4 mm) posteriorly. D and E, Multiple expansile mixed solid and lytic lesions in both iliac bones and L5 vertebral body. F, A lytic lesion of T11 vertebral body.

A 58-year-old woman with history of renal stones was evaluated after her chest radiograph incidentally revealed left pleural lesions (Fig. 1A). Computed tomography revealed multiple mixed sclerotic and lytic expansile lesions in the left eighth, ninth, and tenth ribs (Fig. 1B and 1C). Similar lesions were seen in the scapula (Fig. 1B), pelvic bones, and vertebra (Fig. 1D and 1E). On laboratory investigations, calcium was 12.6 mg/dL (3.15 mmol/L) (normal range [NR], 8.6-10.2 mg/dL [2.15-2.55 mmol/L]), phosphate was 4.4 mg/dL (1.42 mmol/L) (NR, 2.51-4.50 mg/dL [0.81-1.45 mmol/L]), and parathyroid hormone (PTH) level was 804 ng/L (16-87 ng/L). Ultrasound neck localized a large parathyroid adenoma at the lower left thyroid lobe. The patient underwent parathyroidectomy and histopathology revealed fat-depleted parathyroid tissue with a solid pattern of predominantly chief cells and focal oxyphil cells, confirming parathyroid adenoma. Confirmation of hyperparathyroidism established the diagnosis of brown tumors (BTs). Postoperative PTH was less than 3 ng/L. Despite the successful parathyroidectomy, the patient later died of an unrelated critical illness. BTs, rare variants of osteitis fibrosa cystica, are nonmalignant tumors of the giant cell family caused by high PTH, resulting in cystic fibrous tumors of the bone [1]. Radiologically often misunderstood as malignant lesions, BTs are diagnosed clinically in hyperparathyroidism as they cannot be distinguished from other giant cell lesions due to histological similarities [2]. This case emphasizes recognition of BTs in primary hyperparathyroidism.

## Abbreviations

BT: brown tumor
PTH: parathyroid hormone
